# Supplementary material for: Patterns of Intron Gain and Loss in Fungi
Source: PLoS Biol. 2004 Nov 30;2(12):e422. doi: 10.1371/journal.pbio.0020422 (PMC532390; doi:10.1371/journal.pbio.0020422)
Supplement: Table S1 — Also available at http://genes.mit.edu/NielsenEtAl/. (4.3 MB ZIP). [file pbio.0020422.st001.zip › NielsenEtAl/html/773.html]

AN5704.1.NCU05008.1.MG03484.1.FG07865.1


```
 CLUSTAL W (1.82) Multiple Sequence Alignments - Introns Inserted


Sequence 1: NCU05008.1	134 aa
Sequence 2: FG07865.1	147 aa
Sequence 3: AN5704.1	137 aa
Sequence 4: MG03484.1	145 aa
Alignment Length: 161 aa
Number Identitical Residues: 69 aa
Alignment Score (without introns) 2830


MG03484.1 	MMFRATALRSAAAS-VARAGAVRPVVSSSAVARLATRRAAPTAWVPRTAAPWTAVRCYAA
NCU05008.1	-MFRTAALTAARVARPAVASAVR-----AGVARPAFVQAVP------KVAAFQAVRFYSA
FG07865.1 	-MFRSAVLRSLRVA--ARPAAVR-----SFAAPRVASVAVP------KIQSFQAVRFYSA
AN5704.1  	-MFRSAVVRSLRAS-VPRA-----VKPQAALQIRSSPVARPAQLAPRFALP--AVRFYSA
          	 ***::.: :  .:  . .       ..:         * *:   .    .  *** *:*

MG03484.1 	AGGLDKPQVEGRIISLLQKFD-~--------------K~VNDPSK0IQPSSHFANDLGLD
NCU05008.1	GGHLKKDEVFSRIAQVLSGFD-~--------------K0VNDPKN0ITETAHFANDLGLD
FG07865.1 	GGALNKDEVEGRIMSLLQGFDK0QLPNSIEAEANVIFK~VNDVSN0IKSSAHFANDLGLD
AN5704.1  	PAGLKKEEVEGRIVNLLKNFD-~--------------K0VNDASK0INGSSHFSNDLGLD
          	 . *.* :* .** .:*. **                * *** .: *  ::**:******

MG03484.1 	SLDTVEVVMAIEE0EFSIEIPDKDADQIHSV1DKAVEYILSQPDA~N
NCU05008.1	SLDTVEVVMAIEE0EFSIEIPDKDADQIHSV~DKAVEYILSQPDA~N
FG07865.1 	SLDTVEVVMAIEE~EFSIEIPDKDADSIHSV1NQAVEYILNQPDA~A
AN5704.1  	SLDTVEVVMAIEE0EFSIEIPDKEADAIHSV1DKAVEYILAQPDA1H
          	************* *********:** **** ::****** ****
```
